# On Numerical and Analytical Investigation of the Effectiveness of Strengthening of Steel Columns—Case Study

**DOI:** 10.3390/ma18245667

**Published:** 2025-12-17

**Authors:** Jacek Szafran, Paulina Świątkiewicz, Paulina Kaszubska

**Affiliations:** 1 Department of Structural Mechanics, Faculty of Civil Engineering, Architecture and Environmental Engineering, Lodz University of Technology, Al. Politechniki 6, 90-924 Łódź, Poland; paulina.swiatkiewicz@p.lodz.pl; 2 Faculty of Civil Engineering, Architecture and Environmental Engineering, Lodz University of Technology, Al. Politechniki 6, 90-924 Łódź, Poland; 237032@edu.p.lodz.pl

**Keywords:** steel structure reinforcement, geometrically and materially non-linear analysis with imperfections, FEM, reinforcement effectiveness, geometrical imperfections

## Abstract

In the context of growing environmental consciousness, the contemporary construction industry is placing significant emphasis on prolonging the functional lifespan of existing infrastructure. In the event of a modification in the utilisation of a building, an augmentation in the loads transferred to individual elements, or a deterioration in the condition of the structure due to wear and tear, it is often necessary to implement measures for structural reinforcement. The present paper sets out an analysis of the effectiveness of strengthening a steel column manufactured from SHS120×120×5. It was posited that four distinct reinforcement variants could be achieved by the implementation of additional stiffening elements through the process of welding. The efficiency analysis was conducted employing two distinct methodologies. The geometrical imperfection method is employed using the IDEAStatiCa Member 25.0.4 software, whilst the analytical method is implemented through the use of guidelines presented in the literature. It was demonstrated that all of the proposed solutions were capable of meeting the required column capacity when the loads were increased. A comparison was made between the values of the critical forces and the members’ stresses, determined by the selected methods. A substantial discrepancy was identified between the critical force values derived from linear buckling analysis and those calculated using elastic Euler theory. The following discourse herein delineates the primary advantages and limitations of the two aforementioned methods.

## 1. Introduction

The utilisation of steel structures in engineering practice is a pervasive phenomenon, attributable to the numerous advantages they offer in comparison to alternative technologies. The primary benefits associated with these structures include their lightweight nature, which is conducive to ease of transportation and installation; their substantial load-bearing capacity; the expeditious nature of their deployment; their minimal reliance on meteorological conditions; their durability; and their resistance to biological and atmospheric factors. Additionally, the flexibility of modifying existing structures is a notable advantage [1,2]. A significant challenge confronting contemporary construction is the mounting pressure to curtail the industry’s contribution to greenhouse gas emissions. There is a pronounced emphasis on achieving climate neutrality, conducting life-cycle analyses of structures, and employing a circular approach with the objective of minimising construction waste [3,4,5]. In [5] the 9R circularity strategy (Refuse-Rethink-Reduce-Reuse-Repair-Refurbish-Remanufacture-Repurpose-Recycle-Recover) for the design and maintenance of steel structures is presented in detail. The publication cites many factors that should be considered in the case of structural reusability, including material deterioration and the need for knowledge about the history of structural steel, particularly historical codes of practice and product standards.

Probably the most popular option for extending the life of building structures, and therefore reducing waste, is to reinforce them [1,6,7,8,9]. In the case of steel structures, which usually can be easily modified, a viable method is to reinforce individual structural components and connections. The most common factors necessitating reinforcement are changes in design criteria (e.g., increased loads), accidental damage to structural elements, wear and tear accumulated during the service life of the structure, or certain structural defects that may arise at the design stage or during construction [1,6,8,10].

The reinforcement of steel structures can be achieved through the addition of steel plates or rods to the cross-section of the member, either by welding [11] or by bolting [8,9,12]. A number of studies have been conducted on the utilisation of carbon fibre-reinforced polymer (CFRP) strips for the purpose of enhancing the flexural capacity of structures, improving local stability, and fortifying both contemporary and historical puddled metallic structures. For further insight, refer to the following references: [13,14,15]. A method of enhancing the strength of a structure that has been proven to be effective involves the modification of its original static scheme, where such modification is feasible. One such example of this would be the introduction of intermediate supports by means of a tie rod system [6]. The distribution of loading to existing or additional members is a process that, when undertaken, enables the structure to function at an optimal level. It is imperative to acknowledge that alterations to the static scheme, such as modifications to the rigidity of a single element or the introduction of supports, exert a significant influence on the distribution of internal forces within the entire structure. Another method of enhancing the durability and resilience of steel structures may be to incorporate concrete [6]. The utilisation of concrete has been demonstrated to enhance the cross-section of the component considerably. This is advantageous in terms of increasing the rigidity of the cross-section. However, it is also responsible for a substantial increase in the weight of the component.

Analysing a reinforced structure and determining the effectiveness of the reinforcement and, consequently, the load-bearing capacity of the structure after its execution is an extremely complex process. This is due to the necessity of consideration of multitude design situations. It should start by determining the primary strength of the structure at the relevant limit states, which forms the basis for further decisions on whether and how to design structural strengthening. A thorough analysis of the structure should then be carried out during the process of the strengthening work. It is very important that the strengthened structure is subjected to as little loading as possible, as the stresses in the structure must be minimised before the strengthening starts. Finally, the design strength of the strengthened structure is determined in order to assess the effectiveness of the strengthening [1].

The determination of the load-bearing capacity and effectiveness of selected structural solutions can be achieved through the implementation of three fundamental methodologies: (a) experimental testing [16,17,18], (b) using numerical analyses based, for example, on the finite element method [8,9,17,18,19,20,21], and (c) performing analytical calculations based on formulae and guidelines available in relevant standards and literature [8,19,22,23]. The undertaking of experimental tests is an expensive process, which primarily consists of the procurement of suitable samples and measurement apparatus, in addition to the payment of fees associated with the execution of tests in a certified laboratory setting. It is frequently a protracted and intricate process, characterised by a considerable degree of logistical complexity. Consequently, its utilisation in routine engineering practice is precluded; it is employed principally to validate the analytical or numerical boundary conditions adopted in calculations and models [17,18]. The utilisation of numerical analyses, based on the finite element method, is undergoing a marked increase in popularity. The advent of high-performance equipment and the proliferation of diverse software solutions have catalysed the evolution of this discipline. In this particular instance, the geometrical imperfection method is a prominent structural analysis technique. The methodology involves the explicit request of global (sway) and local (bow) imperfections in the programme, followed by the execution of a second-order analysis. The results of this analysis demonstrate a close approximation to the actual response of the structure to the given loads, as illustrated by stress or deformation distribution diagrams. The complexity of this method is predicated on the necessity to accurately model the structure (including connection details) and to determine the appropriate value and direction of imperfections, which exerts a significant influence on the results obtained. This is a subject of interest to a significant number of researchers seeking to establish a systematic approach to the loading of various structural systems that exhibit imperfections [24,25]. The analytical method based on calculations carried out in accordance with guidelines and formulae presented in standards (e.g., Eurocodes) and literature remains the most basic and easiest to implement. This method does not necessitate the use of specialised equipment or the capacity to operate complex calculation programmes. Consequently, the results can be readily compared and analysed. Nevertheless, this approach is widely regarded as being more cautious. A substantial complication for the analysis of steel-reinforced construction is the absence of a European standard dedicated to this purpose. The guidance provided in the standard [26] is applicable exclusively to newly designed elements. In consideration of the aforementioned points, designers utilise formulae obtained from extant literature and implement adaptations to the standard provisions, informed by their own experiential knowledge, intuitive understanding, and construction expertise.

Szafran et al. [8] conducted a comparative analysis of the efficacy of reinforcing the legs of a telecommunication steel lattice tower. This analysis was performed based on standard guidelines and numerical analyses. The analytical approach adopted proved to be very conservative. According to this approach, the existing reinforcement did not enhance the load-bearing capacity of the structure. However, numerical analyses demonstrated that the load-bearing capacity of the reinforced leg is approximately 35–48% higher than that of its unreinforced counterpart.

Numerical analyses of the reinforcement of telecommunication tower cross-braces made of channel sections, as outlined in paper [9], have indicated that the maximum spacing of connections between branches of the near-branch section, as adopted on the basis of standard guidelines for newly designed elements, may be too conservative and frequently unfeasible to meet in the case of reinforced structures.

Prokop et al. [22] sought to ascertain the precision of analytical calculations undertaken on the basis of standards [26,27], employing the outcomes of numerical analyses conducted in ANSYS 17.2. A comprehensive review of the flexural and compression elements made of IPE and RHS was conducted to ascertain the accuracy of the analytical solutions. The findings revealed that the precision of these solutions was contingent on various factors, including buckling modes, the nature of the loading cases, and the slenderness ratio.

As demonstrated in [23], an analysis is provided of the norm approach [28] in relation to experimental and numerical results for composite steel columns composed of four square tubes. It has been demonstrated that the standard [28] is conservative in its assessment of the load capacity of built-up columns absent connectors. Furthermore, it has been established that the conservatism of the standard increases in proportion to the length of the column.

In [19], a method for reducing stiffness in the context of the assessment of flexural-torsional buckling of steel beam-columns was presented. This method was verified by numerical analyses using the geometrical imperfection method. The findings of this study demonstrated that the proposed method yielded results that were notably more precise for irregular and multi-span beams than the outcomes of calculations based on the standard [26]. This enhancement in accuracy can be attributed to the incorporation of the effect of plasticity development on the response of steel elements. It is important to note that the method of stiffness reduction does not necessitate the utilisation of sophisticated software; rather, it is applicable with conventional elastic structural analysis software.

The works [19,22,23] focus on comparing the load-bearing capacity determined by various methods, referring mainly to newly designed, unreinforced structural elements. Conversely, publications [8,9] present an analysis of the compliance of selected provisions of the standard for newly designed elements [26] with the results of numerical analyses, for elements of steel telecommunication towers reinforced in a rather non-standard way. It is important to acknowledge the distinct nature of telecommunication towers as structures, given their substantial height and load characteristics.

This article compares the results obtained using the numerical and the analytical approach for a reinforced column located inside a building. The analysis encompassed four distinct reinforcement variants, involving the incorporation of additional stiffening elements through welding techniques. This solution is a common one in engineering practice, employed to enhance the load-bearing capacity of steel columns. The adopted model incorporated the appropriate level of preload that would affect the column during the reinforcement process. The numerical analyses were performed using IDEA StatiCa Member 25.0.4 software. The analytical approach adopted in this study was in accordance with the guidelines for determining the load-bearing capacity of reinforced elements presented by Zamorowski and Gremza in [1]. The results obtained by both methods were then subjected to a detailed comparison, and the resulting conclusions and findings were presented in the three final chapters of the paper.

## 2. The Subject of the Analyses

### 2.1. The Column in Its Existing Condition

**Figure 1:**
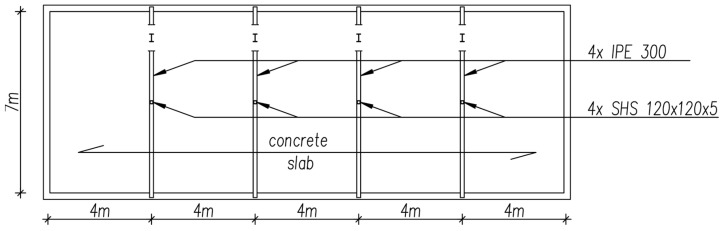
Plan view of the building.

The subject of the analyses carried out in this paper was the internal column in a two-storey commercial and industrial building (Figure 1). The load-bearing structure of the building is composed of steel columns measuring SHS 120 × 120 × 5. The columns, with a height of 6 metres, provide internal support for steel beams measuring IPE300, with a span length of 3.5 m. The outermost supports for these beams are the external walls of the building. The slab, composed of reinforced concrete, is supported by the beams. The columns and beams have been constructed from S235 steel.

It is hypothesised that the loads transferred to the columns will increase due to the planned change in the live load category. For the purpose of the analyses carried out, it was assumed that at the time of reinforcement, the compressive force acting on the column would be 150 kN, while its increase after the change in the value of use loads was estimated at 100 kN (see Table 1).

It is evident that the load on the column would be increased by almost 100% and the ultimate limit of this structural element would be exceeded. It is therefore imperative that reinforcement is implemented.

### 2.2. Reinforcement Options

Four different ways of strengthening the column by welding stiffeners along the length of the column were analysed as:

(A) four L-sections L 30 × 30 × 5;
(B) two C-sections CE80;
(C) two I-sections IPE80;
(D) eight L-sections 20 × 20 × 3.

The stiffening elements in each variant were distributed in such a way that the position of the centre of gravity of the section remained constant. This is of particular significance in the context of reinforcing compression members. In the event of a change in the position of the centre of gravity, the member must be analysed as eccentrically compressed. Consequently, additional bending moments must be incorporated into the calculations.

Table 2 shows the cross-sections through the unreinforced column and reinforced columns together with their geometric parameters–cross-sectional area (A) and moments of inertia relative to the z and y axes of the cross-section (I_z_ and I_y_).

## 3. CBFEM Analysis

A series of numerical analyses were conducted, with the utilisation of the IdeaStatiCa Member 25.0.4 software. The software founded upon the Component-Based Finite Elements Method (CBFEM) [29,30] employed an imperfection approach. The software incorporates key parameters deemed essential for an accurate evaluation of element stability. These parameters encompass the stiffness of the connections, the influence of elements connected to the analysed element (i.e., related members), and material and geometric nonlinearities [31]. The elements under consideration, along with connections, are modelled through the utilisation of quadrilateral shell elements, which possess four nodes and six degrees of freedom at each node. The membrane and flexural components of the deformation are divided. The model incorporates elements inspired by the general MITC4 quadrilaterals by Dvorkin and Bathe [32] with reference to Mindlin’s plate theory [33] to model flexure, while the membrane behaviour of the elements is derived from the work of Ibrahimbegovic [34]. The related members are divided into two parts: the stub part adjacent to the analysed member and the simplified part of the rest of the related member. The stub is modelled as a shell element (full CBFEM model), while the simplified part is modelled as a 1D beam element with six degrees of freedom [31].

### 3.1. Main Assumptions of Analyses

For each of the models created, a three-stage analysis consisting of (a) Materially Non-Linear Analysis (MNA), (b) Linear Buckling Analysis (LBA), and (c) Geometrically and Materially Non-Linear Analysis with Imperfections (GMNIA) was performed. The scope of applicability and the assumptions of the individual analysis steps are presented in [8].

All analyses for reinforced column models were additionally carried out in two configurations:

- preload, which is the case corresponding to the column at the time the strengthening was performed. The compressive force acting on the column during the reinforcement installation work, equal to 150 kN, was introduced as the load on the column in this variant. At this stage of the analysis, IdeaStatiCa Member neglects the effect of the stiffeners—an unreinforced column is analysed.
- target design load, i.e., the case corresponding to a reinforced column under the target design load (compression force of 250 kN). At this stage, the effect of the stiffening elements on the stiffness of the system and on the stress distribution is considered.

It is evident that the elements analysed in this study exhibit a significant degree of slenderness. Consequently, the results of the Material Nonlinear Analysis merely served as a foundation for further analysis. The results of the Linear Buckling Analysis included a 3D visualisation of the mode of loss of stability of the columns, as well as the value of the critical factor α_cr_ for each form of buckling. This critical factor α_cr_ is expressed as the ratio of the elastic critical force (F_cr_) to the value of the load introduced as an input parameter of the analysis (N). The critical force for each model was determined based on the critical coefficient, as demonstrated in the following relation:

(1) $$F_{cr}=\alpha_{cr}\;N$$

The results of the GMNIA analyses included the values and distribution of equivalent stresses in the individual model elements and connection plates. These served directly to assess the effectiveness of the individual strengthening methods. At this stage, the values of the bow local imperfections of the column were taken in accordance with the standard [26] for the plastic analysis of the element. For the unreinforced element and for the reinforced elements for the “preload” variant, the imperfection value was estimated as ${\;e}_{0}=L/250=24\;mm$, which corresponds to the buckling curve “a”, while for the target load of the reinforced columns the imperfection value was calculated as$\;e_{0}=L/150=40\;mm$, i.e., as for the buckling curve “c”. In each case, the imperfection value was assigned to the form of loss of stability with the smallest critical load factor.

For each stage, the analysis was carried out in an iterative manner. The load was gradually increased until the full load set in the input parameters of the analyses was reached, or until the plastic deformation limit of 5% as recommended by the standard [35] was exceeded.

All analyses were carried out taking into account the following material parameters of S235 grade steel [26]:

- nominal value of yield strength $f_{y}=235\;N/{mm}^{2}$
- modulus of elasticity E=210 GPa
- Poisson’s ratio ν=0.3

In the analyses taking into account material non-linearities (MNA and GMNIA), an elastic-plastic model of the steel was adopted with a nominal slope of the yield plateau equal to tan-1(E/1000), where E is the modulus of elasticity of the steel [31]. In contrast, a perfectly elastic steel model was used in the LBA.

### 3.2. Calculation Models

Five calculation models were created-one unreinforced column model (model 0) and four reinforced column models corresponding to the different reinforcement options (models A–D).

#### 3.2.1. Unreinforced Column Model

**Figure 2:**
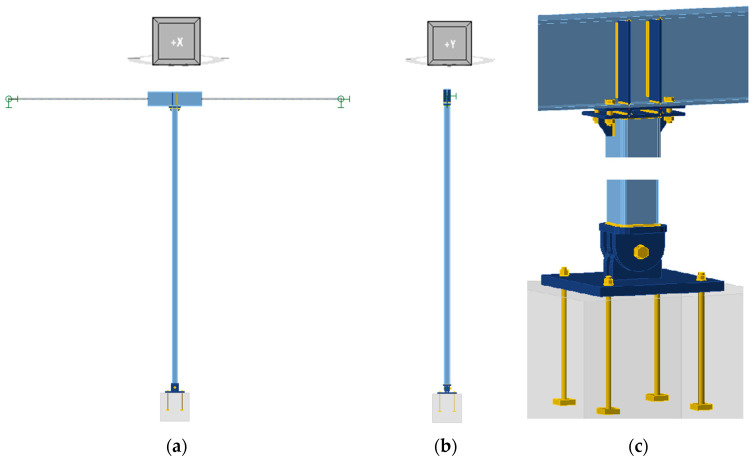
Computational model of the analysed column-Model 0: (**a**) general view in the YZ plane, (**b**) general view in the XZ plane, and (**c**) details.

Figure 2 illustrates the general layout of Model 0, highlighting the connection details between the column and both the beam and the foundation.

The connection between the column and the beam (Figure 2c) was modelled using four M16 class 8.8 bolts. The thickness of the column face plate was assumed to be 10 mm. Furthermore, additional 8 mm thick ribs were modelled within the beam, welded between the I-beam flanges with 3 mm fillet welds on both sides. Additionally, stiffening ribs with a thickness of 6 mm were modelled between the column and the end plate. The connection to the base plate (Figure 2c) was modelled as a pinned connection using an M24 cl. 8.8 pin. The bottom plate of the column and the vertical plates were assumed to be 20 mm thick, while the base plate was assumed to be 30 mm thick. The connection of the base plate to the foundation was modelled using 4 M16 cl. 8.8 anchors with washer plates. For modelling contact between plates, the standard penalty method was incorporated. The penalty stiffness is controlled by a heuristic algorithm during nonlinear iteration as described in [29]. Whereas bolts were modelled by a dependent nonlinear spring explained in detail in [29], for both the bolts connecting the column to the beam and to the base plate, it was assumed that the shear plane passes through the threaded part of the bolt. The modelling of these connections enabled the consideration of their stiffness in the element analyses. The load-bearing capacity of these connections was not the subject of the analyses carried out and was not tested. The beam was modelled as a related member with an overall length of 7 m in the direction of the Y-axis of the global coordinate system of the model. The model incorporates boundary conditions at both extremities of the beam that correspond to a non-articulated joint. These boundary conditions are characterised by locked displacements along three perpendicular axes and released rotations about these axes. The column, beam and connection plates were modelled as manufactured from S235 steel.

**Figure 3:**
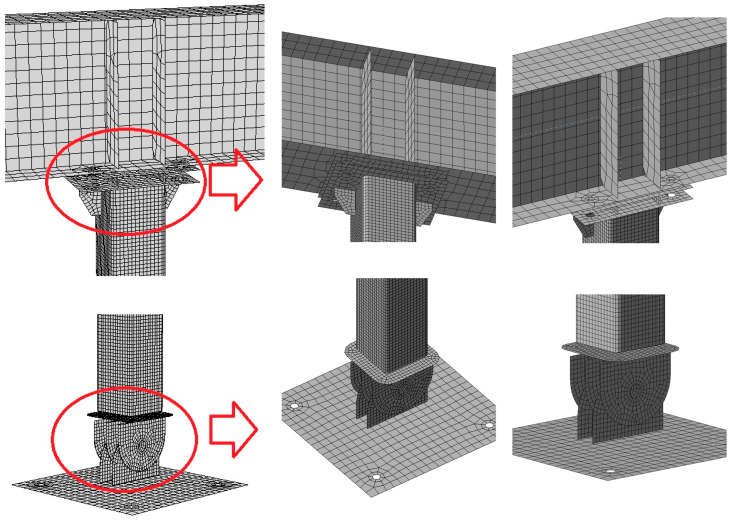
Computational model of the analysed column-Model 0-mesh details.

The meshing employed in numerical analyses is illustrated in Figure 3. The maximum size of the element in the mesh created was 50 mm, with the number of elements on the SHS wall set at 16, and 12 for the webs and flanges of the other elements.

#### 3.2.2. Reinforced Column Models

**Figure 4:**
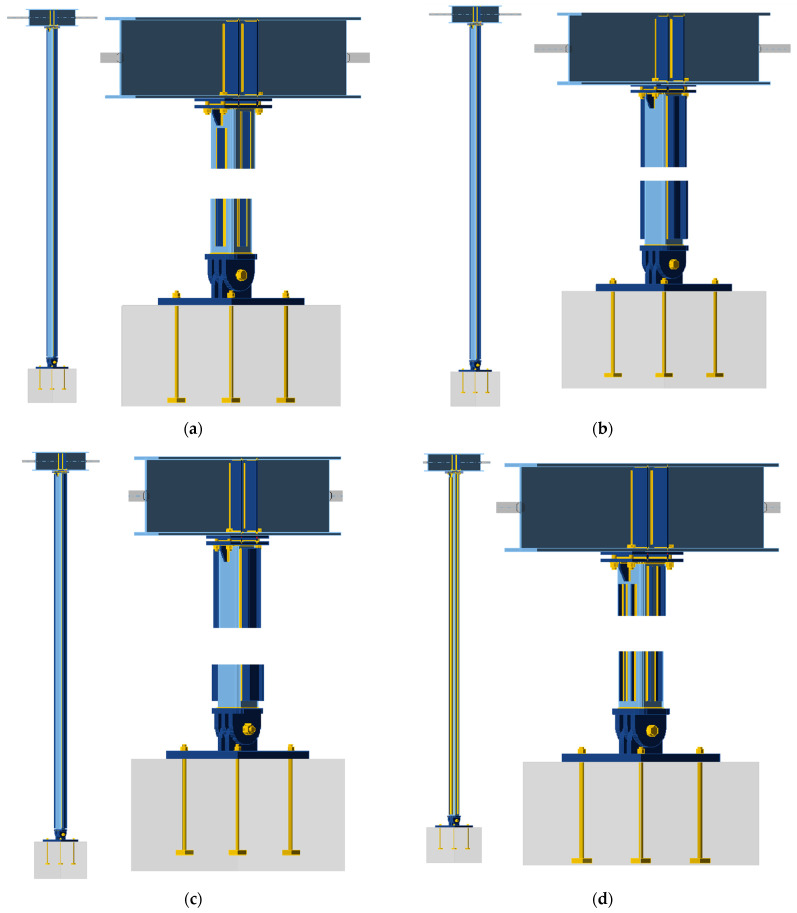
Computational models of the reinforced column with details of connections to the beam and the foundation: (**a**) Model A, (**b**) Model B, (**c**) Model C, (**d**) Model D.

The general view and connection details of the reinforced column design models for the four reinforcement variants are shown in Figure 4. The reinforced column (SHS120×120×5) is represented by the colour light blue, the added stiffeners are indicated by dark blue, and the welds and bolts are denoted by yellow. It is evident that the parameters pertaining to the column-to-foundation and beam connection, in addition to the beam boundary conditions and finite element mesh parameters, remained unaltered from those of the unreinforced model. Additional elements were modelled as welded to the column using 3 mm fillet welds. Due to concerns regarding the execution of the reinforcement, it was not feasible to connect the stiffeners directly to the column-to-beam and column-to-foundation connection plates.

### 3.3. Results of Analysis for Unreinforced Column

#### 3.3.1. LBA

**Figure 5:**
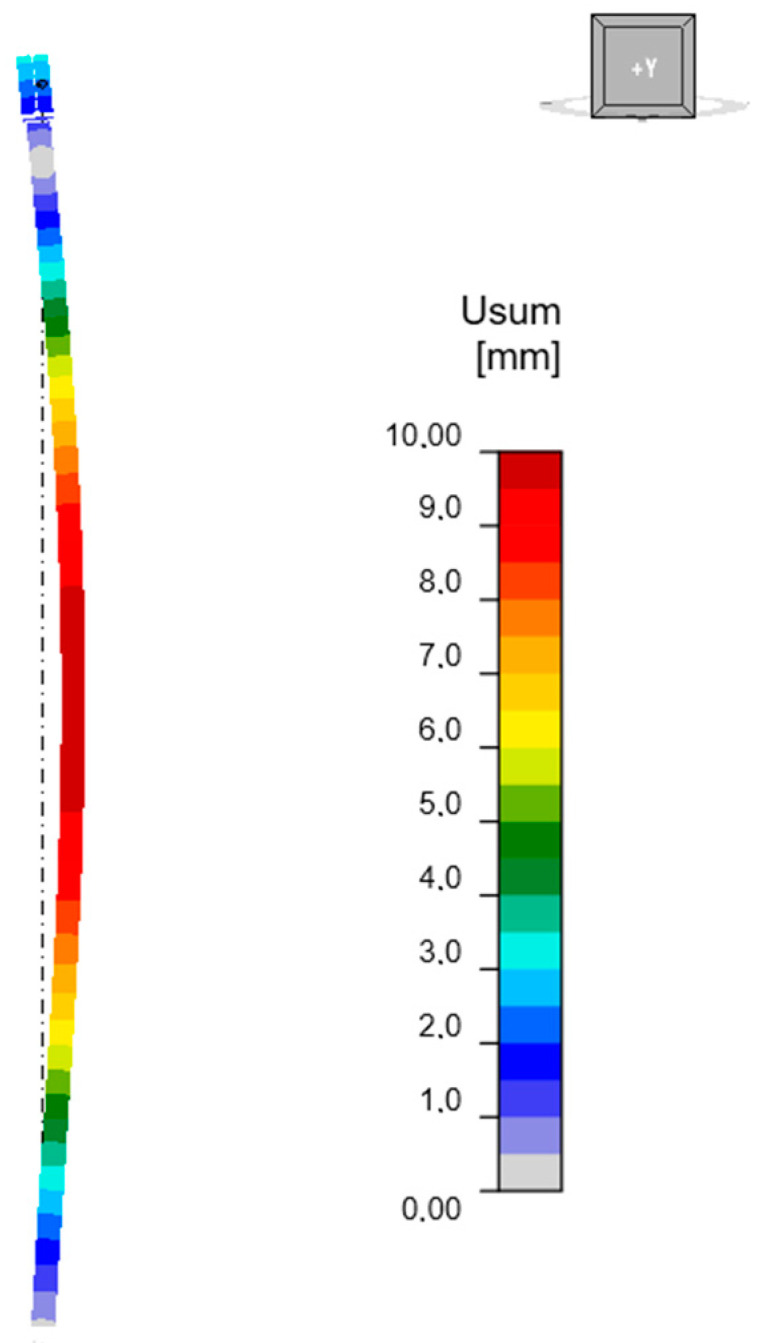
First form of unreinforced column instability.

The mode of the first form of loss of stability for an unreinforced column is shown in Figure 5. The buckling occurs in the X-axis direction of the global coordinate system. The corresponding critical buckling factor$\;\alpha_{cr}$ for a compression force of 250 kN is equal to 1.70, so the estimated critical force is 425 kN.

#### 3.3.2. GMNIA Analysis

The GMNIA analysis was discontinued at 97.7% of the design load of the column after the change in live load category (250 kN) due to the plastic strain limit being exceeded. The greatest stresses and deformations occurred in the connection between the column and the foundation, as well as in the middle of the column’s span. This finding serves to substantiate the hypothesis that the column lacks sufficient load-bearing capacity, thus necessitating the implementation of reinforcement measures.

### 3.4. Results of Analyses for Different Reinforcement Options

#### 3.4.1. LBA

The first forms of loss of stability for the reinforced models are outlined in Table 3. In models A and D, where the additional elements are distributed pointwise symmetrically with respect to the centre of gravity (i.e., where the moment of inertia of the composite section with respect to both axes is the same), buckling occurs in the direction of the X axis of the global coordinate system of the model. This is equivalent to the behaviour of an unreinforced column. This is the direction perpendicular to the direction of the beam supported by the columns. Conversely, for models B and C, buckling occurs in the Y-axis direction of the global coordinate system. This phenomenon can be attributed to the substantial disparity in stiffness relative to the local axes of the columns in these models. LBA indicates that buckling occurs around the weaker axis of the section, despite the presence of beam and the increased stiffness of the connection to the beam in this direction. These disparities are deemed to be of pivotal significance when conducting a thorough analysis of the critical force and buckling resistance of the elements.

As illustrated in Table 4, the values of the critical coefficients obtained for the design target load case (250 kN) are summarised, in addition to the critical forces calculated from these coefficients for Models A–D. It is evident that the buckling coefficients and, consequently, the critical forces for all variants are considerably higher than the value obtained for the unreinforced column variant. The most significant increase in the critical force compared to the unreinforced component was observed in variant B, i.e., reinforcement employing two CE80 channels.

#### 3.4.2. GMNIA Analysis

**Figure 6:**
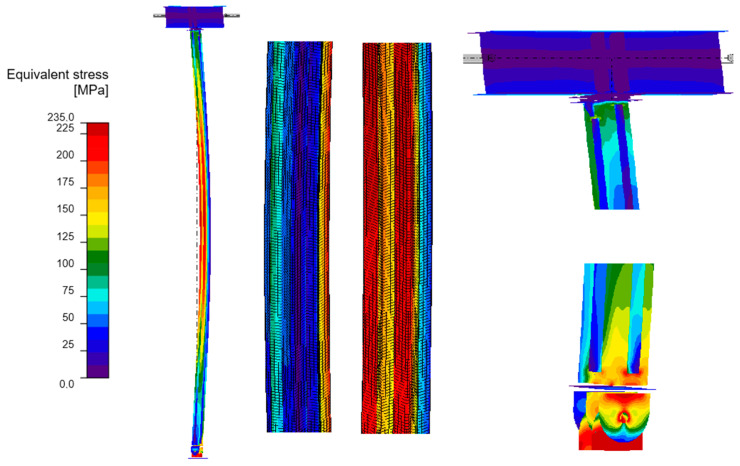
Equivalent stresses from GMNIA analysis—Model A: whole structure (**on the left**), close-ups on the centre of the column (**in the middle**) and close-ups on the connections (**on the right**).

**Figure 7:**
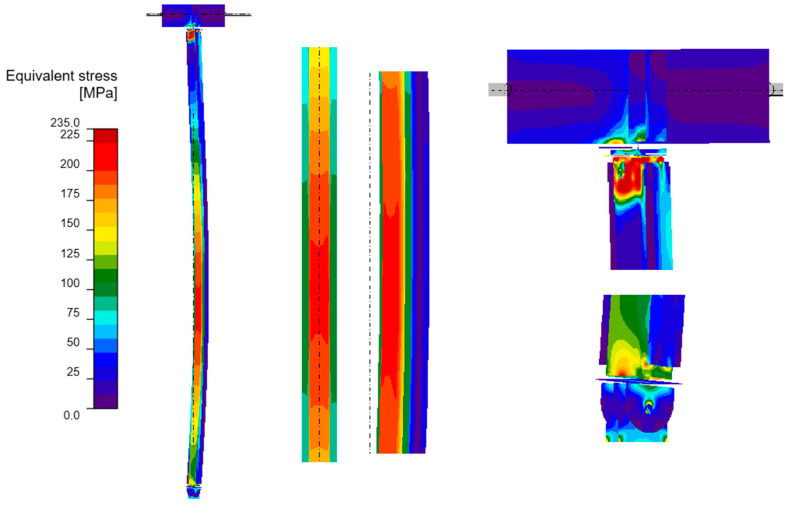
Equivalent stresses from GMNIA analysis—Model B: the whole structure (**on the left**), close-ups on the centre of the column (**in the middle**) and close-ups on the connections (**on the right**).

**Figure 8:**
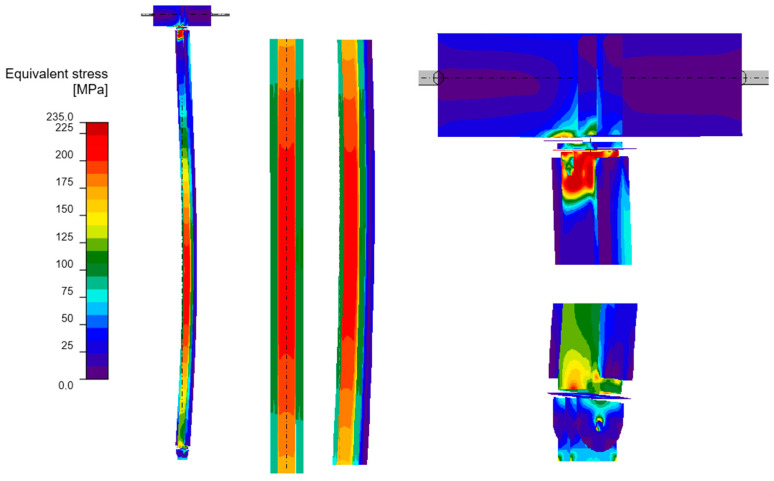
Equivalent stresses from GMNIA analysis—Model C: the whole structure (**on the left**), close-ups on the centre of the column (**in the middle**) and close-ups on the connections (**on the right**).

**Figure 9:**
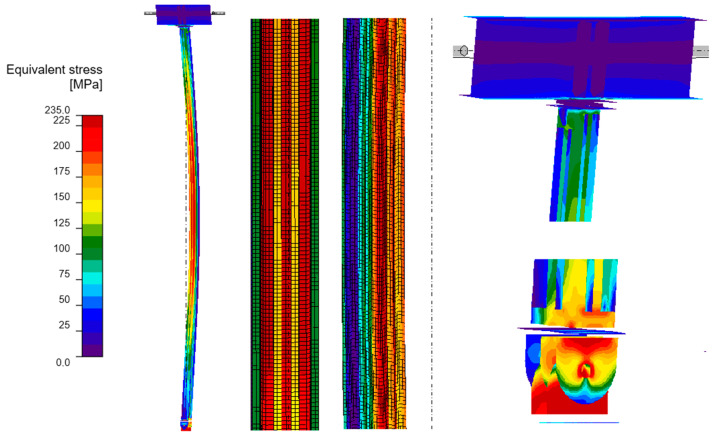
Equivalent stresses from GMNIA analysis—Model D: the whole structure (**on the left**), close-ups on the centre of the column (**in the middle**) and close-ups on the connections (**on the right**).

Maps of equivalent stresses obtained using GMNIA analysis for models A–D are presented in Figure 6, Figure 7, Figure 8 and Figure 9. Each figure is composed of two images: a general view of the column, and close-up views of the central part and the connections. As demonstrated in the close-up images of models A and D, mesh refinement around the centre of the column facilitates the distinction between the SHS120×120×5 core and the welded elements. A significant disproportion of the stress values calculated in the original element (SHS) and in the reinforcing elements is evident in all the models. The phenomenon under discussion is the result of the preload, which is applied to the column prior to the implementation of reinforcement. A further factor that contributes to the stress disproportion is the absence of technical capability to connect the stiffening elements directly to the connection plates. These aspects have been shown to have a significant impact on the effectiveness of the reinforcement.

It is imperative to note the locations of local stress concentrations for the individual models, which manifest particularly in the vicinity of the joints. For models A and D, the critical stresses appear to be located at the base plate connection, where the vertical plates connected by pin are bent. Conversely, for models B and C, the maximum local stresses are observed in the connection to the beam in the proximity of the stiffening ribs welded to the column. The disparity in the locations of local stress concentrations is attributable to the fact that models B and C exhibit a buckling perpendicular to models A and D. Irrespective of the location of these local stress concentrations, they pose a threat to the safety of the structure and are challenging to discern with simplified analytical calculations. It is evident that numerical methods facilitate the acquisition of more detailed information, thereby enabling the identification of areas necessitating meticulous consideration, reinforcement, or redesign.

As demonstrated in Table 5, the maximum equivalent stresses in all models reached a value around the yield strength of the steel. Notably, model A, which employed a reinforcing configuration involving four angles, yielded the lowest stress levels. This observation suggests that this particular reinforcing configuration may offer optimal effectiveness.

## 4. Analytical Approach

The analytical method for assessing the stability of a compression member is based on the assumptions presented in Section 6.3 of the standard [26]. In this method, bow imperfections of the elements are taken into account by assigning an appropriate buckling curve and calculating the buckling factor of the element. It is imperative that calculations encompass the potential for buckling in various planes, with consideration given to the influence of diverse boundary conditions and cross-section parameters. In the case of frame structures, the flexibility of the supporting nodes must be taken into consideration with regard to the effective stiffness of the adjoining elements [36]. A comprehensive discourse on the stability concerns of metal structures is elucidated by Rykaluk in [37]. It is generally hypothesised that the analytical method is more accessible to the average engineer than the geometrical imperfection method, as it does not require specialised software, but only a proper evaluation of the buckling length.

However, it should be noted that the standard [26] only covers components that have been newly designed. At the present time, there is no European standard that specifies precise guidelines for determining the resistance of steel-reinforced members. The literature does not provide a clear definition of how to take into account preload, nor of the implementation of connections between reinforced and reinforcing elements in order to ensure adequate effectiveness of the solution. Given the prevalence of research in the field of reinforced steel structures, researchers have sought to determine analytical formulae. In this paper, the formulae and relationships presented in [1] are employed to calculate the load-bearing capacity of reinforced columns using the analytical method.

The analytical calculations assumed the material parameters of the steel (yield strength, modulus of elasticity, Poisson’s ratio) to be unchanged from the numerical analyses. The columns were calculated under the assumption of pinned support on both sides. In this section of the analysis, the determination of the compliance of nodes and approaching elements was eschewed, as the objective was to ascertain whether the simplified engineering approach would exhibit a substantial error in comparison with the geometrical imperfection method. It was hypothesised that great emphasis would be placed on speed, and that a reduced level of computational complexity would be possible.

### 4.1. Resistance of an Unreinforced Axially Compressed Member

According to the Eurocode [26] the structure under compression must satisfy the following conditions:

(2) $$N_{Ed}/N_{c,Rd}\leq 1\;and\;N_{Ed}/N_{b,Rd}\leq 1.$$

They comprise the design resistance of the cross-section subjected to uniform compression $\;N_{c,Rd}$, as well as the design resistance of the compression member with consideration of buckling (e.g., [38,39,40]), which is given by the following formula [26]:

(3) $$N_{b,Rd}=\chi \;A\;f_{y}/\gamma_{M1}.$$

where χ is the reduction factor that incorporates an imperfection factor α for the relevant buckling curve and the slenderness of the member $\bar{\lambda };\;A$ is the cross-sectional area; ${\;f}_{y}$ is the yield strength of the steel, while ${\;\gamma }_{M1}$ is the safety factor adopted equal to 1.0.

The SHS section 120×120×5 was classified as a class 1 section in accordance with the standard [26]. Conversely, the “a” curve, which is applicable to hot-rolled tubular sections, was adopted as the buckling curve [26]. The values of the parameters and formulae necessary to determine the buckling resistance of an unreinforced column are summarised in Table 6, while the results are presented in Table 7. It is evident that, given the column cross-section is symmetrical and the buckling length factor was deliberately dispensed with respect to both axes, all parameters are identical irrespective of the axis (y or z) considered in calculations. Consequently, Table 7 does not present a distinction between axes.

The strength criterion presented in Formula (2) is not fulfilled as$\;N_{Ed}/N_{b,1,Rd}>1$. Therefore, the structure needs reinforcement.

### 4.2. Resistance of the Reinforced Axially Compressed Member

Based on [1], the resistance condition of a reinforced steel bar without additional bending and torsion is assumed as:

(4) $$\frac{N_{1,Ed}}{N_{b,1,Rd}}+\frac{{∆N}_{Ed}}{N_{b,2,Rd}}\leq 1,$$

where $\;N_{1,Ed}$ is the design value of the axial force applied to the member before strengthening, ${∆N}_{Ed}$ is the additional axial force after strengthening, whereas $\;N_{b,1,Rd}$ and $\;N_{b,2,Rd}$ are the design values of the buckling resistance of a compressed member before and after strengthening, respectively. $\;N_{b,1,Rd}$ is calculated using initial parameters of the structure according to Equation (3), whereas $\;N_{b,2,Rd}$ takes the following forms [1]:

- the buckling resistance of a reinforced compressed member under assumption that it buckles as one member:

(5) $$N_{b,2,Rd}=\chi_{2}\cdot \frac{\;A_{1}\;f_{y,1}+A_{2}\;f_{y,2}}{\gamma_{M1}},$$

where $\chi_{2}$ is the buckling reduction factor calculated after reinforcement when the initial member and the additional parts are treated as a whole; $A_{1}$ and $\;f_{y,1}$ are the cross-sectional area and the yield strength of initial column, whereas $\;A_{2}\;$and${\;f}_{y,2}$ refer to values of the cross-sectional area and the yield strength of added elements. If a member is Class 4 the effective area of a cross-section $A_{eff}$ should be considered in calculations.

- the buckling resistance of a reinforced compressed member when initial member and additional parts buckle independently
  (6) $$N_{b,2,Rd}=\chi_{1}^{\prime }\cdot \frac{\;A_{1}\;f_{y,1}}{\gamma_{M1}}+\chi_{2}^{\prime }\cdot \frac{\;A_{2}\;f_{y,2}}{\gamma_{M1}},$$
  where $\;\chi_{1}^{\prime }$ and where $\;\chi_{2}^{\prime }$ are the buckling reduction factors for the primary member and added members, respectively. If due to strengthening of the member the boundary conditions are changed, new conditions should be included in computations.

The approach delineated above is predicated on the summation of the stresses from the original force acting on the original section and the additional load acting on the extended section. In the case under consideration, it is assumed that the additional stiffeners will be welded to the column. Consequently, independent buckling of the SHS 120 × 120 × 5 and its additional elements is rendered impossible. This assertion is corroborated by the buckling forms obtained in the LBA conducted in the preceding section of this paper. It is evident that Equation (5) was utilised in the calculations undertaken in this study.

The approach documented in [1] incorporates the impact of preload on reinforcement efficiency. Furthermore, the aforementioned formulae permit the utilisation of steel as the reinforcing element, with the possibility of employing distinct material parameters, such as the yield strength. This is of particular significance in the context of the reinforcement of structures that have been in existence for decades, for which there is an absence of comprehensive archival documentation to assess the grade of steel, and whose material parameters may have undergone degradation to a certain extent over time. It is important to note that the formulae cited do not specify the technical solutions (e.g., how the reinforced and reinforcing elements are connected to each other, how the reinforcing elements are connected to the nodes) that should be used for the formulae to be valid. The responsibility for selecting and implementing the optimum design solutions falls upon the structural strengthening engineer.

The calculation of the buckling coefficient for a reinforced member utilises the formulae summarised in Table 8, which are analogous to those for an unreinforced column. In the case of reinforced columns, the buckling curve “c” was adopted as the most unfavourable possibility for elements that are not thin-walled.

Table 9 shows buckling resistance of the reinforced compressed member treated as a whole calculated according to Equation (5) with respect to the indicated axis. Values of $\;N_{b,2,Rd}$ refer to calculations of the buckling reduction factor $\;\chi_{1}$, that include joint geometric parameters of initial cross-section and cross-sections added during reinforcement. Values of critical load$\;N_{cr}$, relative slenderness of the member $\;\bar{\lambda }$, parameter  ϕ and reduction factor $\;\chi_{1}$ obtained according to [26] are also given in the table to enable tracking the differences between models. The most unfavourable reduction factor for each reinforcement type is marked in bold, and the buckling load capacity $\;N_{b,2,Rd}$ includes this value.

### 4.3. Evaluation of Effectiveness of Selected Reinforcement Variants

The obtained buckling load capacities (Table 7 and Table 9) and the design values of loads (Table 1) included in Formula (4) lead to the information whether bearing capacity condition is fulfilled after reinforcement. The results are presented in Table 10 together with cross-sectional area increment calculated as ratio of the cross-sectional area of the reinforced element to the initial cross-sectional area, second moment of inertia increment (minimum ratio of the moment of inertia of the strengthening elements to the moment of inertia of initial cross-section). Additionally, bearing capacity increment was computed according to formula: $\left(N_{b,Rd,2}-N_{b,Rd,1}\right)/N_{b,Rd,1}\times 100\%$.

As demonstrated in Table 10, the proposed reinforcement types have been shown to be effective in ensuring the adequate strengthening of the initial column. It is evident that the most efficient distribution of additional cross-section, with regard to the bearing capacity increment in relation to the mass increment ratio, is observed in the case of reinforcement D. For this particular case, there was an increase in bearing capacity of over 25%, accompanied by a mass increment of almost 40%. As can be seen from the data, a similar but slightly worse effectiveness is obtained in reinforcement A. The last two reinforcement (B and C) types are characterised by a much worse bearing capacity increment to mass increment ratio.

## 5. Comparison of Results

It can be concluded from the findings of both the geometrical imperfection method (numerical analyses) and the analytical method (calculations according to [1]) that an unreinforced column will be incapable of bearing the increased load. In contrast, the load-bearing conditions of the element are fulfilled for all the reinforcement variants analysed.

However, it is important to note the differences in the results of the individual analysis steps for the two methods. A synthesis of critical force values for both unreinforced and reinforced columns derived from both numerical analyses and analytical calculations is presented in Table 11. It is evident that the critical force values for the numerical analyses exceed those for the analytical calculations. This phenomenon can be attributed, in part, to the variation in the implementation of boundary conditions. IdeaSatiCa Member performs automated calculations and incorporates the stiffness of the connections and related elements in the LBA. In the course of the analytical calculations, a significant simplification was adopted, with the element being considered as an ideal, non-deformable bar with pinned supports on both ends. A salient observation is that the value of the elastic Euler critical force (analytical calculation) is proportional to the moment of inertia of the section. It has been demonstrated that such a relationship is not observed for the critical forces that are obtained from numerical analyses. The critical forces for models B and C are larger than for models A and B, despite the smaller moments of inertia.

The observed phenomenon can be attributed to the fact that the buckling of models A and D occurs in a plane perpendicular to the buckling plane of models C and D. It is evident that the stiffness of the entire system–that is to say, the stiffness of the member, the connections and the related elements–is not uniform in both planes. For models A and D, where the moments of inertia are equal about both axes, the stiffness of the connections and of the beam connected to the column have a decisive influence on the buckling direction, so that buckling occurs in the direction perpendicular to the beam. Conversely, for models C and D, the buckling plane is determined by the substantial disparity in section stiffness. The buckling phenomenon occurs around the weak axis of the section, which is parallel to the beam. In order to ensure consistency between analytical and numerical results, it is necessary to determine and take into account the stiffness of the joints in both planes when employing the analytical method to determine the buckling length. This process necessitates the execution of complex and time-consuming calculations. The problem would not occur if the system and its constituent elements in both planes exhibited equivalent stiffness.

The comparison of the load-bearing capacity of reinforced columns for the analytical method is significantly less complex than for the geometrical imperfection method. As demonstrated in Table 9 and Table 10, it is possible to determine which variant exhibits the most favourable ratio of increase in resistance to increase in cross-sectional area (and consequently in element mass). Subsequent analysis indicated that variants A and D were the most favourable in this aspect. It is important to note that the numerical analyses do not directly provide a value for the load-bearing capacity of the element; rather, they represent the state of stress and deformation under a given load. However, this enables a comprehensive understanding of how the structure responds to a specific load. It was found that the lowest stresses occurred for Variant A. For all models, the stress value in the reinforced element was higher than in the additional stiffening elements. This finding indicates the limited effectiveness of the reinforcement.

It is evident that numerical analyses offer a distinct advantage in terms of providing information regarding local stress concentrations. This facilitates the identification of areas necessitating special attention or even redesign to ensure the safety of the structure. In some cases, a relatively uncomplicated alteration in the geometry or thickness of plates can result in a substantial increase in the load-bearing capacity. In the context of analytical method calculations, the designer is required to identify these locations through the application of personal experience and intuition.

## 6. Discussion

This study employed two distinct methods to assess the load-bearing capacity of the reinforced element. The findings indicate that all the proposed reinforcement options will provide adequate load-bearing capacity for the column and ensure safe load transfer. However, a closer inspection reveals that the detailed results of both analyses are not fully consistent. The results of the analytical method indicate that reinforcement variants A and D are the most favourable. These variants are distinguished by their exceptional critical force and resistance to buckling. However, numerical analyses yielded divergent results, with models B and C exhibiting the highest critical forces, and model A demonstrating the lowest stresses. The analytical method revealed a proportional relationship between the component’s resistance and the critical force. The numerical analyses do not appear to corroborate this relationship. Furthermore, an analysis of the equivalent stress distributions obtained in IdeaStatiCa Member reveals discrepancies in stress levels between the reinforced element and the reinforcing elements. The stresses in the additional stiffening elements are lower than in SHS120×120×5, indicating a limited efficiency of force transfer between these elements. A further aspect that must be given full consideration in the definitive selection of reinforcement options is the intricacy of the welding process. It is evident that as the length of the welds increases, the cost of executing the reinforcement also increases. The impact of welding stresses is also of increasing significance; however, this was not considered in the analyses conducted.

A significant benefit of the conventional approach in structural analysis is the expeditious nature of calculation and the ease with which it can be accessed. The analysis can be conducted without the necessity for specialist software; only access to standard formulae is required. In the case of the geometrical imperfection method, implementation is characterised by a significant barrier to entry. The procurement of specialised technical equipment and licensed software is a prerequisite for undertaking this endeavour. Furthermore, it is imperative to attain proficiency in advanced operational and validation procedures within the selected computational environment. This is essential to ensure the minimisation of numerical errors and the attainment of high accuracy in the results obtained. However, it should be emphasised that the analytical method presented in the standard [26] is intended only for newly designed components. In practice, this signifies that there are currently no precise European guidelines for the analysis of reinforced steel members. In this situation, calculations are based on literature formulae, which, however, are also somewhat limited in scope. For instance, the formulae presented in the paper [1] do not explicitly correspond to a particular method of connecting reinforcing and reinforced elements. It appears evident that the implementation of an additional element through welding would result in a more substantial increase in the load-bearing capacity when compared with the utilisation of bolts for connection to the original element. In addition, it should be noted that the buckling curves presented in [26] do not encompass curve fitting for complex elements, such as those examined in the present study. In view of the aforementioned points, in order to facilitate applying analytical method in assessing the load-bearing capacity of reinforced steel members, it is essential that a new European standard dedicated to reinforced steel structures is developed and introduced. It is imperative that the standard incorporates provisions to account for the divergent material parameters of reinforced and reinforcing elements, as well as the varied methods through which additional elements are connected to the original element. It is imperative that the standard incorporates the technical intricacies involved in the reinforcement of existing structures, such as constrained access to the reinforced element or the necessity for reinforcement during the structure’s operational life.

The load-bearing capacity values obtained through calculations employing the analytical method facilitate straightforward comparison of the results across the range of options under consideration. In the case of the geometrical imperfection method, the evaluation of the results is more complex and must be undertaken through a rigorous analysis of the stress distribution, as well as the shape and size of the deformation. However, by presenting the results in this manner, it is possible to observe a greater number of phenomena and identify potential problems. The utilisation of numerical analyses, employing the geometrical imperfection method, facilitates the acquisition of information pertaining to various parameters, including but not limited to local stress concentrations.

The effectiveness of strengthening a compression member is contingent upon the distribution of the additional material, that is to say, the increase in cross-sectional area and the moment of inertia of the cross-section. Concurrently, the objective should invariably be to distribute the supplementary elements in as symmetrical a manner as possible (with regard to both axes). However, it should be noted that this is not always feasible. In extant constructions, access to all sides of the section may be obstructed by technical equipment, finishes or other fittings. The pivotal consideration is therefore to introduce the supplementary components in such a manner that the position of the centre of gravity remains unaltered and no additional bending is introduced into the compression members (unless this has been factored into the calculations). In such situations, a detailed analysis of the stiffness of the entire system in both planes must be carried out. In software based on the geometrical imperfection method, which generally automatically takes into account the stiffness of the nodes and related elements, the determination of the critical buckling plane and the critical load factor is left to the calculation algorithms. However, it is imperative to exercise caution when incorporating the model, as the generated finite element mesh may influence the precision of the results obtained. The experience of the designer and the ability to analyse the results obtained are essential to assess the correctness of the calculations. The analytical method is a complex process due to the number of parameters that must be taken into consideration during the calculation process. Errors in the estimation of the stiffness of supports, additional elements or bracing can result in erroneous calculations of buckling length and incorrect resistance values. Another significant aspect that must be considered is the introduction of reinforcing elements to the connections at the ends of the reinforced member, where technically feasible. This has a beneficial effect on the stress distribution.

It is important to note that the analyses conducted do not allow for a definitive conclusion regarding the relative conservatism of the methods. The validity of this assertion is contingent upon the particular design case under consideration, and the precision with which the designer has executed the analyses. In the case of elements that are relatively stocky in nature and composed of elementary cross-sections, exhibiting uniform stiffness in both directions (i.e., both cross-sectional stiffness and supports are consistent), it is likely that the analytical method will prove to be adequate. Conversely, in the context of slender and more complex structures, the analytical method is an effective criterion for the preliminary selection of a limited number of favourable solutions, which are then analysed using the geometrical imperfection method.

## 7. Conclusions

The objective of the analyses conducted in this study was to compare the geometric imperfection approach and analytical methods when assessing the effectiveness of steel column reinforcement. A particular focus was placed on the discrepancies in the results, the intricacy of the analyses, the time-consuming nature of the process, and the ease with which the results obtained can be analysed and conclusions drawn. The geometrical imperfection method analyses were conducted in IdeaStatiCa Member 25.0.4, while the analytical calculations were based on the guidelines presented in [1]. The results of the critical forces obtained for both methods were then compared with each other. An analysis was also conducted on the load capacity values, as well as on the values and distribution of equivalent stresses.

The analytical calculations and numerical analyses carried out allowed the following conclusions to be drawn:

- It is asserted that all variants analysed will ensure the safe transfer of increased loads. With regard to load-bearing capacity, variant A is demonstrably superior when a comparison is made between the results obtained from both methods. Nonetheless, prior to determining a definitive solution, it is imperative to consider the expenses associated with workmanship and welding stresses, which have the potential to influence the load-bearing capacity of the element.
- The reinforcement efficiency of elements in compression is a complex function, depending on both the increase in cross-sectional area and the increase in moment of inertia. It is imperative that the reinforcement elements are positioned in as symmetrical manner as possible with respect to the centre of gravity of the cross-section, thereby facilitating optimised stress distribution.
- It is evident that both the analytical and geometrical imperfection methods possess a range of advantages and limitations. The geometrical imperfection method necessitates the acquisition of licensed software and the attainment of proficiency in its utilisation. However, it should be noted that this approach enables the finite stiffness of the nodes to be incorporated into the analysis in an automatic manner. Furthermore, it facilitates the identification of local stress concentrations, thereby providing a response of the structure to a given load that is proximate to the actual response. Conversely, the analytical method is characterised by its expeditiousness and accessibility, both in terms of implementation and interpretation. The tool is to be regarded as a highly effective instrument for preliminary comparative assessment. However, given the necessity of incorporating the rigidity of nodes and the intricacies of stress states, undertaking analytical method necessitates complex calculations. The assumption of the buckling length and plane is of crucial importance.
- It is not possible to provide a definitive answer to the question of which method (analytical or imperfection) exhibits a greater degree of conservatism. The result of the analysis is contingent on the designer’s assumptions and the modelling strategy adopted for the structure. The accuracy of the results obtained by means of the geometrical imperfection method is directly proportional to the precision of the joint modelling, the correctness of the implementation of geometric imperfections, and the density and quality of the finite element mesh. The cornerstone of the analytical method is the accurate evaluation of the boundary conditions and the precise determination of the buckling length and buckling plane of the element.
- It is recommended that a hybrid approach be employed in the case of structures exhibiting significant slenderness and complex static schemes, whereby both methods are applied simultaneously. The analytical method has been demonstrated to be a successful approach for the preliminary selection and comparison of the effectiveness of different structural solutions. Subsequently, the selected variants should be analysed in detail using the geometrical imperfection method. This will allow the ultimate load capacity to be estimated and the actual response of the structure to applied loads to be observed.
- The application of the analytical method in the analysis of reinforced structures is contingent upon the introduction of a new European standard. The new standard should be dedicated solely to the analysis of reinforced steel structures and should contain detailed guidelines and analytical formulae for various reinforcement scenarios.

## Figures and Tables

**Table 1:** Design values of loads applied to the analysed column.

Force	Notation	Value
Initial axial force	$N_{1,Ed}$	150 kN
Additional axial force after reinforcement	${∆N}_{Ed}$	100 kN

**Table 2:** Parameters of the cross-section of the unreinforced and reinforced columns.

		Parameters of the Cross-Section		
No.	Geometry	*A* [cm^2^]	I_(z)_ [cm^4^]	I_(y)_ [cm^4^]
0	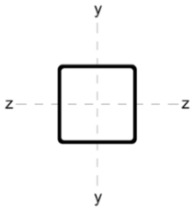	22.70	498.00	498.00
A	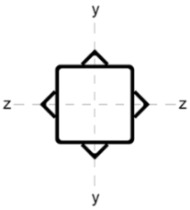	33.81	784.47	784.47
B	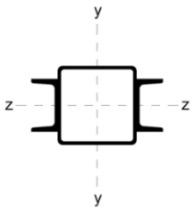	40.66	676.80	1483.31
C	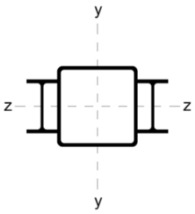	37.99	658.28	1568.09
D	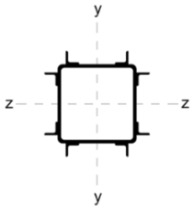	31.68	774.16	774.16

**Table 3:** First form of buckling Model A, Model B, Model C, Model D.

	Model A	Model B	Model C	Model D
Form of buckling	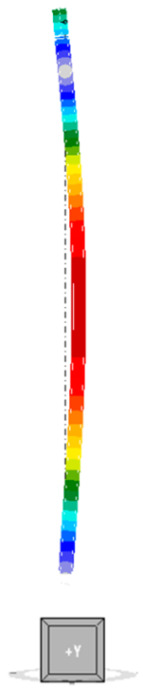	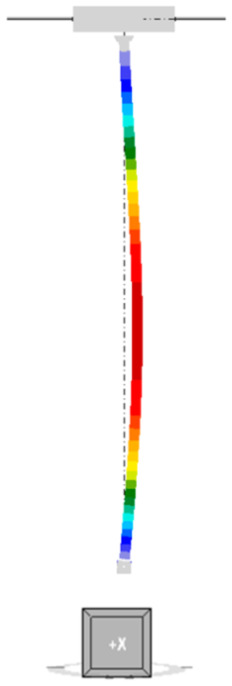	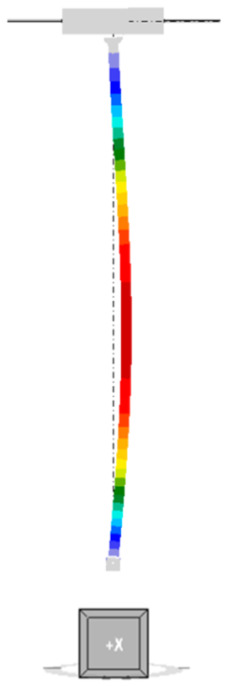	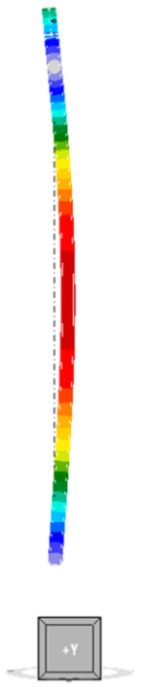
Direction of buckling	Buckling in the X direction of the model’s global coordinate system	Buckling in the Y direction of the model’s global coordinate system	Buckling in the Y direction of the model’s global coordinate system	Buckling in the X direction of the model’s global coordinate system

**Table 4:** Linear buckling analysis results.

Model	$\mathrm{Critical}\;\mathrm{Buckling}\;\mathrm{Factor}\;\alpha_{cr}$ [-]	$\mathrm{Critical}\;\mathrm{Force}\;F_{cr}$ [kN]
A	2.42	605.0
B	2.60	650.0
C	2.55	637.5
D	2.27	567.5

**Table 5:** GMNIA results-maximum stresses.

Model	Maximum Stresses [MPa]
A	227.7
B	236.7
C	236.9
D	235.0

**Table 6:** The basic parameters used in calculating the buckling capacity of the column before reinforcement.

Parameter	Notation	Value
A	cross-sectional area	22.70 cm^2^
f_y_	yield strength of steel	235 MPa
γ_M1_	safety factor	1.0
χ	reduction factor	$1*{\left(\phi +\sqrt{\phi^{2}-{\bar{\lambda }}^{2}}\right)}^{-1}$
ϕ	parameter	$0.5*[1+\alpha *\left({\bar{\lambda }}^{2}-0.2\right)+{\bar{\lambda }}^{2}]$
α	imperfection factor of the buckling curve a	0.21
$\bar{\lambda }$	relative slenderness	$\sqrt{A*\;f_{y}*{\left(N_{cr}\right)}^{-1}}$
$N_{cr}$	Euler buckling critical force	$\pi^{2}*E*I*{\left(\mu *L\right)}^{-2}$
μ	buckling length coefficient	1.0
E	modulus of elasticity	210 GPa
I	moment of inertia of a cross-section	498 cm^4^
L	length of the analysed element	6.0 m

**Table 7:** Buckling resistance of the initial compression member.

Cross-Section	$N_{cr}$ [kN]	$\bar{\lambda }$ [-]	α [-]	ϕ [-]	χ [-]	$N_{b,1,Rd}$ [kN]	$N_{Ed}/N_{b,1,Rd}$ [-]
SHS 120×120×5	286.71	1.364	0.21	1.553	0.436	232.55	1.075

**Table 8:** The basic parameters used in calculating the buckling capacity of the reinforced column.

Parameter	Notation	Value
A	cross-sectional area	individual for each reinforcement method (Table 2)
f_y_	yield strength of steel	235 MPa
γ_M1_	safety factor	1.0
χ	reduction factor	$1*{\left(\phi +\sqrt{\phi^{2}-{\bar{\lambda }}^{2}}\right)}^{-1}$
ϕ	parameter	$0.5*[1+\alpha *({\bar{\lambda }}^{2}-0.2)+{\bar{\lambda }}^{2}]$
α	imperfection factor of the buckling curve c	0.49
$\bar{\lambda }$	relative slenderness	$\sqrt{A*\;f_{y}*{\left(N_{cr}\right)}^{-1}}$
$N_{cr}$	Euler buckling critical force	$\pi^{2}*E*I*{\left(\mu *L\right)}^{-2}$
μ	buckling length coefficient	1.0
E	modulus of elasticity	210 GPa
I	moment of inertia of a cross-section	individual for each reinforcement method (Table 2)
L	length of the analysed element	6.0 m

**Table 9:** Buckling load capacity of the reinforced member treated as a whole-$N_{b,2,Rd}.$

Reinforcement	Axis	$N_{cr}$ [kN]	$\bar{\lambda }$ [-]	ϕ [-]	$\chi_{1}$ * [-]	$N_{b,2,Rd}$ [kN]
A	y	451.64	1.326	1.656	**0.378**	300.24
	z	451.64	1.326	1.656	**0.378**	
B	y	853.98	1.058	1.270	0.507	281.00
	z	389.65	1.566	2.061	**0.294**	
C	y	902.79	0.994	1.189	0.543	270.96
	z	378.99	1.535	2.005	**0.305**	
D	y	445.71	1.293	1.603	**0.329**	291.88
	z	445.71	1.293	1.603	**0.329**	

* buckling reduction factor calculated on basis of joint geometric parameters of the initial and added cross-sections.

**Table 10:** Comparison of reinforcement variants’ effectiveness regarding mass increment and bearing capacity increment.

Reinforcement Variant	Cross-Sectional Area Increment [%]	Moment of Inertia Increment [%]	Bearing Capacity Condition (Equation (3)) [-]	Bearing Capacity Increment [%]
A	48.94	57.52	0.927	29.11
B	79.12	35.90	0.964	20.83
C	67.36	32.18	0.984	16.52
D	39.56	55.45	0.944	25.51

**Table 11:** Comparison of critical forces obtained using the numerical and analytical methods.

Model	Moment of Inertia Corresponding to the Decisive Form of Buckling	Critical Force from Numerical Analyses Numerical Analysis N_cr,FEM_	Euler Elastic Critical Force from Analytical Calculations N_cr,Euler_	N_cr,FEM_/N_cr,Euler_
	**[cm^4^]**	**[kN]**	**[kN]**	**[-]**
0	498.00	425.0	286.71	1.48
A	784.47	605.0	451.64	1.34
B	676.80	650.0	389.65	1.67
C	658.28	637.5	378.99	1.68
D	774.16	567.5	445.71	1.27

## Data Availability

The original contributions presented in this study are included in the article. Further inquiries can be directed to the corresponding author.
